# Editorial: Sports medicine and physical rehabilitation, volume III

**DOI:** 10.3389/fvets.2025.1551208

**Published:** 2025-02-19

**Authors:** David Levine, Arielle Pechette Markley, Denis J. Marcellin-Little, Heidi Reesink

**Affiliations:** ^1^Department of Physical Therapy, University of Tennessee at Chattanooga, Chattanooga, TN, United States; ^2^Red Sage Integrative Veterinary Partners, Fort Collins, CO, United States; ^3^Department of Surgical and Radiological Sciences, University of California, Davis, Davis, CA, United States; ^4^Department of Clinical Sciences, College of Veterinary Medicine, Cornell University, Ithaca, NY, United States

**Keywords:** veterinary rehabilitation, rehabilitation, sports medicine, orthopedics, canine rehabilitation, equine rehabilitation, veterinary sports medicine, neurology—dog

Veterinary *Sports Medicine and Physical Rehabilitation* is now a well-established field. The articles in the first and second volumes of the Veterinary *Sports Medicine and Physical Rehabilitation* ebooks, published by Frontiers in Veterinary Science, have been viewed more than 175,000 times and have been downloaded more than 32,000 times.

This Research Topic is the third volume in the series and includes 15 research articles by 50 authors focusing on sports medicine (seven articles) and physical rehabilitation (eight articles).

Two articles focused on the management of orthopedic problems in horses. A study by Hallowell et al. evaluated the systemic absorption of triamcinolone after intrasynovial (antebrachiocarpal) and extrasynovial (sacroiliac) injection. The study also evaluated the effects of triamcinolone absorption on glucose, insulin, cortisol, and adrenocorticotropic hormone. Intrasynovial injections led to increased plasma triamcinolone levels 8 to 36 h after injection. Insulin and glucose were also elevated after injection. No adverse reactions were noted and all horses had normal physical examination parameters throughout the study period. This study suggests that more work is needed to investigate risk factors for corticosteroid-associated laminitis in horses. A study by Gruyaert et al. evaluated the proximity of needles placed for palmar digital nerve blocks to nearby synovial structures using computed tomography. The majority of (97%) needles avoided synovial penetration, but the risk of synovial penetration was higher when needle placement was distal and when synovial structures were distended. Clinicians should be aware that there is a low risk of inadvertent penetration of the distal interphalangeal joint, navicular bursa, or digital flexor tendon sheath when performing palmar digital perineural anesthesia.

Thirteen articles focused on the management of orthopedic or neurologic problems in dogs. A 45-day clinical trial by Talsma et al. evaluated the efficacy of cannabidiol in dogs with mobility disorders and assessed the safety of the cannabidiol in combination with a non-steroidal anti-inflammatory drug (NSAID). Subjective outcome measures, including client questionnaires and blinded veterinary assessments, improved over the course of the study, but greater ALP elevations were seen in dogs receiving combination therapy with an NSAID. Further research is needed to better understand the safety and efficacy of CBD when administered with NSAIDs and other medications.

Two rehabilitation articles evaluated extracorporeal shockwave therapy. A clinical trial by Joseph et al. compared the noise reactivity and tolerance to treatment between a standard and a novel trode designed to reduce the peak focal energy used to deliver the extracorporeal shockwave therapy. Dogs tolerated the novel trode better, allowing the delivery of 10% more shocks at a 20% higher energy level. In a case series, Tsai and Alvarez described the response to extracorporeal shockwave therapy and physical rehabilitation in working dogs with fibrotic myopathy. On average, dogs were able to return to work for ~3 years after the time of diagnosis, a more favorable outcome than the outcome described in the previous case series.

A study by Blake et al. described the kinetic parameters of dogs walking across cavaletti rails at heights of 5, 10, 15, and 20 cm (2, 4, 6, and 8 inches). Increasing cavaletti rail height resulted in a decrease in gait velocity and an associated increase in gait cycle duration. A clinical study by Schwartz et al. evaluated gluteal and quadriceps femoris muscle activation using surface electromyography in dogs recovering from hemilaminectomy compared to normal dogs. Muscle activation was increased by more than 30% in the operated dogs compared to normal dogs and was 30% less on the operated side than on the unoperated side. The greater muscle activity in the group that underwent hemilaminectomy may be explained by hypertonia, but making comparisons of EMG findings between dogs is inherently problematic because of potential differences in electrode placement.

Narum et al. surveyed 1,221 caretakers of dogs using an assistive mobility cart to evaluate quality of life, function, and adverse events. With the use of a mobility cart, ~60% of caretakers reported improvement in the quality of life of their dog or cat. More than 60% of the animals experienced an adverse event and half of those were wounds. This study was the largest of its kind and provided helpful information to pet caretakers and clinicians. A study by Christie et al. evaluated the agreement in body condition and muscle condition when a group of working dogs was evaluated by one handler and three veterinarians. Handlers appeared to have a higher likelihood of rating their dogs as optimally conditioned and muscled than veterinarians. This project emphasized the importance of standardizing training and guidelines for the assessment of body and muscle condition in working dogs.

Six articles focused on sporting dogs. A retrospective study by Hattendorf et al. summarized the injuries sustained by ~1,000 Alaskan and Siberian husky sled dogs during a 1,000-mile (1,600-km) sled dog race. Approximately half of the dogs had a medical problem during the race and one-third sustained an orthopedic injury. Carpal and shoulder injuries were most common. Five studies focused on agility dogs. One survey study by Alva et al. evaluated the outcome of perceived limb injuries in agility dogs. Owners sought veterinary care more than 80% of the time and often sought specialty care. Rest was the most common treatment used. Two-thirds of the dogs returned to sport within 3 months of perceived injury, although dogs with stifle injuries reported a longer time to return to sport. A survey study by Kieves et al. evaluated risk factors for stifle injuries in agility dogs. Five factors were associated with an increased risk of stifle joint injury: being heavier, being a Border Collie, being a male and neutered before 10 months of age, being a female and spayed before the first heat cycle, having an 18- to 24-year-old handler, and teeter contact behavior. The biomechanics of agility dogs negotiating the teeter, a dynamic agility contact obstacle were further evaluated in a study by Pechette Markley, Wood, et al.. In this study, dogs used various strategies to negotiate the teeter. The study provided insight into these strategies, opening the door for further research focused on dynamic stability and postural control. Another study by Pechette Markley, Kieves, et al. investigated the effects of the ground surface (dirt, grass, or sand) on speed during agility trials. Trials run on sand were slower than trials run on grass or dirt. This study highlights the need for kinetic and kinematic studies comparing surfaces in agility events and how these may relate to injury risk. A study by Sellon et al. described the methodology for developing a rating of perceived exertion (RPE) tool to help quantify training load in agility dogs. RPE is widely used in the training of human athletes and this research adapted the tool for use in agility dogs. The tool was quick and easy to use but requires validation.

Like the first two volumes, this third ebook volume on *Sports Medicine and Physical Rehabilitation* will be a valuable resource for veterinary rehabilitation and sports medicine professionals. The Editors are pleased with the strength and diversity of the 15 articles included in this volume. They provide valuable information for veterinary sports medicine and physical rehabilitation practitioners and provide a foundational context for future research in the field.

